# Artificial intelligence in orthodontics and orthognathic surgery: a bibliometric analysis of the 100 most-cited articles

**DOI:** 10.1186/s13005-023-00383-0

**Published:** 2023-08-23

**Authors:** Ka Fai Wong, Xiang Yao Lam, Yuhao Jiang, Andy Wai Kan Yeung, Yifan Lin

**Affiliations:** 1 Division of Paediatric Dentistry and Orthodontics, Faculty of Dentistry, the University of Hong Kong, Prince Philip Dental Hospital, No.34 Hospital Road, Hong Kong SAR, China; 2 Department of Restorative Dentistry, Faculty of Dentistry, the National University of Malaysia, Kuala Lumpur, Malaysia; 3 Division of Oral and Maxillofacial Radiology, Applied Oral Sciences and Community Dental Care, Faculty of Dentistry, the University of Hong Kong, Hong Kong SAR, China

**Keywords:** Artificial intelligence, Bibliometrics, Orthodontics and orthognathic surgery

## Abstract

**Background:**

The application of artificial intelligence (AI) in orthodontics and orthognathic surgery has gained significant attention in recent years. However, there is a lack of bibliometric reports that analyze the academic literature in this field to identify publishing and citation trends. By conducting an analysis of the top 100 most-cited articles on AI in orthodontics and orthognathic surgery, we aim to unveil popular research topics, key authors, institutions, countries, and journals in this area.

**Methods:**

A comprehensive search was conducted in the Web of Science (WOS) electronic database to identify the top 100 most-cited articles on AI in orthodontics and orthognathic surgery. Publication and citation data were obtained and further analyzed and visualized using R Biblioshiny. The key domains of the 100 articles were also identified.

**Results:**

The top 100 most-cited articles were published between 2005 and 2022, contributed by 458 authors, with an average citation count of 22.09. South Korea emerged as the leading contributor with the highest number of publications (28) and citations (595), followed by China (16, 373), and the United States (7, 248). Notably, six South Korean authors ranked among the top 10 contributors, and three South Korean institutions were listed as the most productive. International collaborations were predominantly observed between the United States, China, and South Korea. The main domains of the articles focused on automated imaging assessment (42%), aiding diagnosis and treatment planning (34%), and the assessment of growth and development (10%). Besides, a positive correlation was observed between the testing sample size and citation counts (*P* = 0.010), as well as between the time of publication and citation counts (*P* < 0.001).

**Conclusions:**

The utilization of AI in orthodontics and orthognathic surgery has shown remarkable progress, particularly in the domains of imaging analysis, diagnosis and treatment planning, and growth and development assessment. This bibliometric analysis provides valuable insights into the top-cited articles and the trends of AI research in this field.

**Supplementary Information:**

The online version contains supplementary material available at 10.1186/s13005-023-00383-0.

## Introduction

Artificial Intelligence (AI) has emerged as a transformative technology in healthcare, with practical applications extending to various fields, including dentistry. The applications of AI in dentistry are extensive, encompassing tasks such as assisting in radiographic interpretation, detecting early signs of dental caries, cysts, and tumors with greater accuracy, assessing growth and development, and predicting treatment outcomes [1, 2]. Specifically, AI holds significant potential in providing invaluable assistance in orthodontics and orthognathic surgery, where precision, accuracy, and reliability are of utmost importance.

Orthodontics and orthognathic surgery require exceptional precision and accuracy, as their outcomes often entail irreversible changes. Therefore, conducting a comprehensive clinical and radiographic examination is crucial to establish an accurate diagnosis [3]. Unlike humans, who require rest and exhibit inconsistencies, AI technology offers the ability to process vast amounts of data with consistent accuracy, making it a powerful tool in the assessment and treatment planning process [4, 5]. By harnessing the power of AI, dentistry can reach unprecedented levels of precision and reliability in orthodontics and orthognathic surgery, ultimately leading to enhanced outcomes for both patients and providers.

Despite in early stages, AI has already made significant strides in the field of orthodontics and orthognathic surgery [5, 6]. Recent scoping reviews identified several key domains within AI research in orthodontics, including diagnosis and treatment planning, automated anatomic landmark detection and analysis, and assessment of growth and development [6, 7]. Most of these AI models are based on either artificial neural networks or convolutional neural networks [5]. However, it remains essential to conduct further investigations to verify the reliability and applicability of AI models [8]. The integration of AI technology with thorough clinical assessment and professional judgment has the potential to enhance workflow efficiency, thereby facilitating effective orthodontic and orthognathic surgery treatment procedures.

With the rapid rise of technology in this field, it is not surprising to observe an increasing research trend of incorporating AI technology in orthodontics and orthognathic surgery. To gain a comprehensive understanding of the publication landscape and citation patterns, a bibliometric study is necessary to qualitatively and quantitatively analyze the publication characteristics of scholarly work focusing on AI in orthodontics. This analysis encompasses parameters such as authorship, countries of origin, institutional affiliations, and other pertinent factors, aiming to shed light on the current research trends in AI for orthodontics and orthognathic surgery [9]. Previous bibliometric and citation analyses in orthodontics have focused on the most-cited articles [10–13], or explored various topics, including temporary anchorage devices [14], clear aligner treatments [15], and lingual orthodontics [16]. The primary aim of this study is to develop a comprehensive profile of research conducted on the application of AI in orthodontics and orthognathic surgery, providing invaluable insights for researchers, clinicians, and other stakeholders. This will help identify essential study topics and trends associated with this field, facilitating the development of more targeted and effective AI-based interventions and enhancing the quality of orthodontic and orthognathic surgery care.

## Material and methods

### Search strategy

A comprehensive electronic literature search was performed in the Web of Science (WoS) Core Collection database on May 30th, 2023, with no initial time restriction. Two independent reviewers conducted the search using the following algorithm: ALL = (orthod* OR cephal* OR craniofacial * OR maxillo*) AND ALL = (deep learn* OR artificial intelligen* OR machine learn* OR convolutional neural network* OR RNN OR CNN* OR Recurrent neural network* OR FCN* OR Fully Convolutional Network* OR artificial neural network*). The search results were then refined to exclude document types other than research articles and reviews, such as editorials, letters, meeting abstracts, and corrections.

The articles were sorted in descending order based on their total citation count. Two independent reviewers conducted a thorough screening of the titles and abstracts of the identified articles. After the screening process, the reviewers compared their results and reached a consensus on the selection of the top 100 most-cited articles in the field of orthodontics and orthognathic surgery, with a Cohen's kappa value of 89.11%.

### Data extraction and analysis

Bibliometric parameters, such as article title, citation count, citation density, year of publication, authorship, country of authors, institution of publication, and keywords, were extracted from the selected articles in the WoS Core Collection. The R Biblioshiny was employed to analyze and visually represent the relevant bibliometric data. The classification of the topics were determined by reviewing the titles and abstracts. Additionally, the research articles were assessed to identify the training and testing sample sizes. Spearman correlation analysis was conducted to examine whether there is any significant correlation between the sample size, years of publication, and citation counts. The analysis was conducted using Statistical Package for Social Sciences (SPSS) software version 26.0. A *P*-value < 0.05 was considered as significant.

## Results

**Fig. 1:**
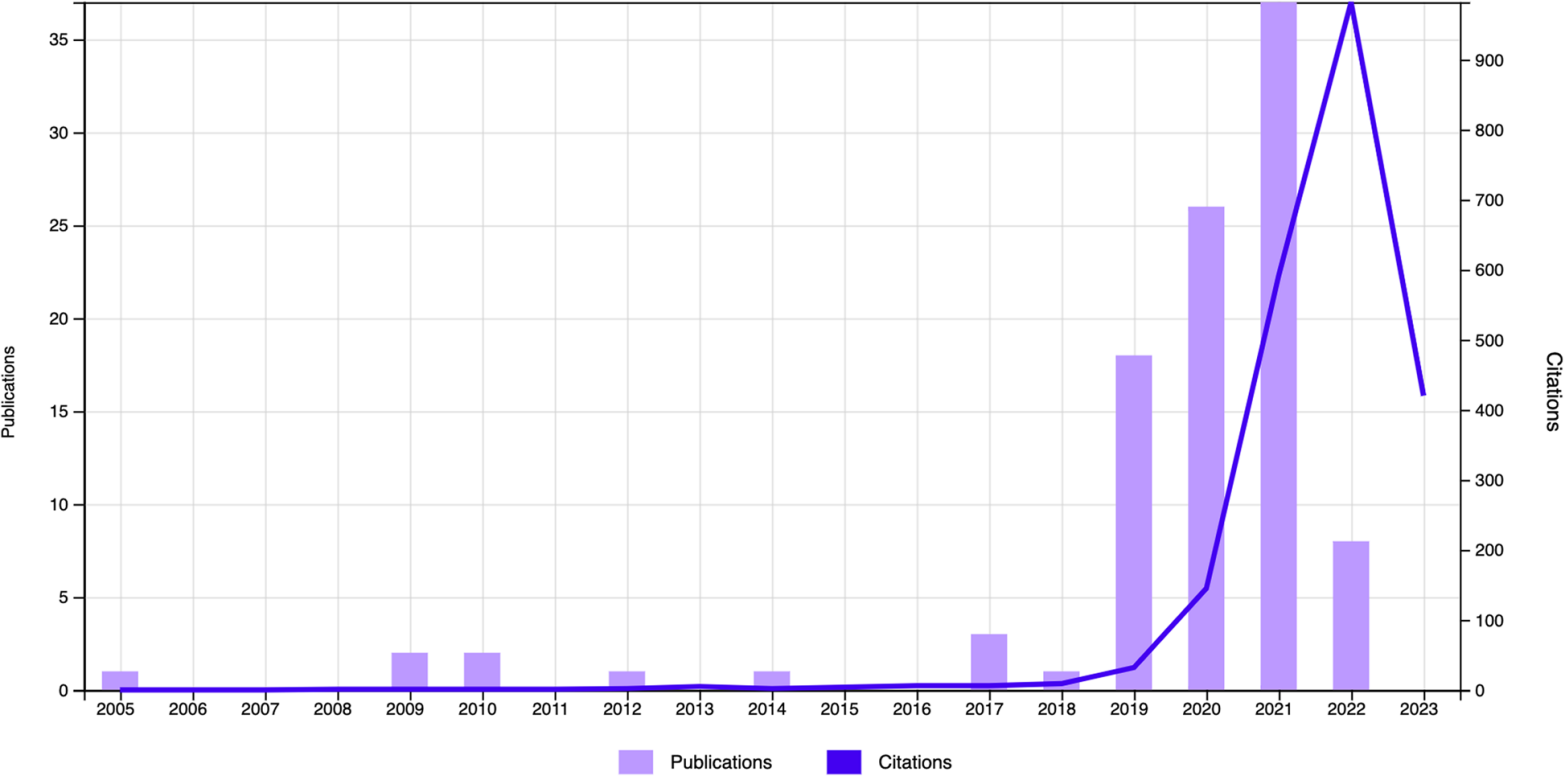
Publication and citation trend of the top 100 most-cited articles. The bars represent the annual publication count and the line indicates the annual citation count

According to the search strategy, a total of 634 articles were initially identified and subsequently filtered based on their relevance to AI, orthodontics, and orthognathic surgery. Documents other than research articles or reviews were excluded. Following this, the filtered articles were arranged in descending order based on their citation count, and the top 100-most cited articles in AI related to orthodontics and orthognathic surgery were identified. The analysis revealed that the top 100 most-cited articles were published between 2005 and 2022. The average citation count per article was found to be 22.09. The publication and citation trend of the 100 articles is depicted in Fig. 1, indicating a rising trend in both publications and citations, particularly during 2019–2021. Notably, among the 100 most-cited articles, 37 of them were published in 2021.

### Most-cited articles and citation correlation analysis

Table 1 displays the top 20 most cited articles pertaining to AI in orthodontics and orthognathic surgery, ranked by their total citation count. Among these, the most-cited AI article titled "Fully automated quantitative cephalometry using convolutional neural networks," authored by Arik et al. in 2017 [17], has accumulated a total of 119 citations. Notably, Kunz et al.'s [18] article published in 2020, titled "Artificial intelligence in orthodontics: Evaluation of a fully automated cephalometric analysis using a customized convolutional neural network," stands out with the highest average citation per year (24.00). The full list of the top 100 most-cited articles can be found in the Supplementary file. The citation correlation analysis revealed that there was no significant correlation between the training sample size and citation counts (correlation coefficient = 0.043, *P* = 0.727). However, a positive correlation was observed between the testing sample size and citation counts (correlation coefficient = 0.283, *P* = 0.010). Additionally, a significant positive correlation was found between the years of publication (2023 – publication year) and citation counts (correlation coefficient = 0.561, *P* < 0.001).

**Table 1:** The top 20 most cited articles in AI in orthodontics and orthognathic surgery

No	Article titles	Authors	Journals	Year of publication	Citations	Average citations per year	Classification of the topics
1	Fully automated quantitative cephalometry using convolutional neural networks	Arik et al.	Journal of Medical Imaging	2017	119	19.83	2. Automated cephalometric landmarking and/or analysis
2	Artificial intelligence in orthodontics Evaluation of a fully automated cephalometric analysis using a customized convolutional neural network	Kunz et al.	Journal of Orofacial Orthopedics	2020	72	24.00	2. Automated cephalometric landmarking and/or analysis
3	Automated identification of cephalometric landmarks: Part 2-Might it be better than human?	Hwang et al.	Angle Orthodontist	2020	65	21.67	2. Automated cephalometric landmarking and/or analysis
4	Artificial Neural Network Modeling for Deciding if Extractions Are Necessary Prior to Orthodontic Treatment	Xie et al.	Angle Orthodontist	2010	65	5.00	1. Diagnosis and treatment planning
5	Automated identification of cephalometric landmarks: Part 1-Comparisons between the latest deep-learning methods YOLOV3 and SSD	Park et al.	Angle Orthodontist	2019	64	16.00	2. Automated cephalometric landmarking and/or analysis
6	Automated Skeletal Classification with Lateral Cephalometry Based on Artificial Intelligence	Yu et al.	Journal of Dental Research	2020	57	19.00	1. Diagnosis and treatment planning
7	Deep Geodesic Learning for Segmentation and Anatomical Landmarking	Torosdagli et al.	IEEE Transactions on Medical Imaging	2019	57	14.25	2. Automated cephalometric landmarking and/or analysis
8	Automated cephalometric landmark detection with confidence regions using Bayesian convolutional neural networks	Lee et al.	BMC Oral Health	2020	49	16.33	2. Automated cephalometric landmarking and/or analysis
9	Applying artificial intelligence to assess the impact of orthognathic treatment on facial attractiveness and estimated age	Patcas et al	International Journal of Oral & Maxillofacial Surgery	2019	47	11.75	1. Diagnosis and treatment planning
10	An Attention-Guided Deep Regression Model for Landmark Detection in Cephalograms	Zhong et al.	Lecture Notes in Computer Science	2019	45	11.25	2. Automated cephalometric landmarking and/or analysis
11	Orthodontic Treatment Planning based on Artificial Neural Networks	Li et al.	Scientific Reports	2019	43	10.75	1. Diagnosis and treatment planning
12	Web-based fully automated cephalometric analysis by deep learning	Kim et al.	Computer Methods and Programs in Biomedicine	2020	42	14.00	2. Automated cephalometric landmarking and/or analysis
13	Personal Computer-Based Cephalometric Landmark Detection With Deep Learning, Using Cephalograms on the Internet	Nishimoto et al.	The Journal of Craniofacial Surgery	2019	42	10.50	2. Automated cephalometric landmarking and/or analysis
14	Usage and comparison of artificial intelligence algorithms for determination of growth and development by cervical vertebrae stages in orthodontics	Kok et al.	Progress in Orthodontics	2019	41	10.25	3. Assessment of growth and development
15	Artificial Intelligent Model With Neural Network Machine Learning for the Diagnosis of Orthognathic Surgery	Choi et al.	The Journal of Craniofacial Surgery	2019	39	9.75	1. Diagnosis and treatment planning
16	Artificial Intelligence for Fast and Accurate 3-Dimensional Tooth Segmentation on Cone-beam Computed Tomography	Lahoud et al.	The Journal of Endodontics	2021	37	18.50	2. Automated cephalometric landmarking and/or analysis
17	A machine learning framework for automated diagnosis and computer-assisted planning in plastic and reconstructive surgery	Knoops et al.	Scientific Reports	2019	37	9.25	1. Diagnosis and treatment planning
18	Cephalometric Landmark Detection by Attentive Feature Pyramid Fusion and Regression-Voting	Chen et al.	Lecture Notes in Computer Science	2019	34	8.50	2. Automated cephalometric landmarking and/or analysis
19	Current Applications, Opportunities, and Limitations of AI for 3D Imaging in Dental Research and Practice	Hung et al.	International Journal of Environmental Research and Public Health	2020	32	10.67	4. Miscellaneous
20	Automatic Cephalometric Landmark Detection on X-ray Images Using a Deep-Learning Method	Song et al.	Applied Sciences	2020	32	10.67	2. Automated cephalometric landmarking and/or analysis

### Journal profile

**Fig. 2:**
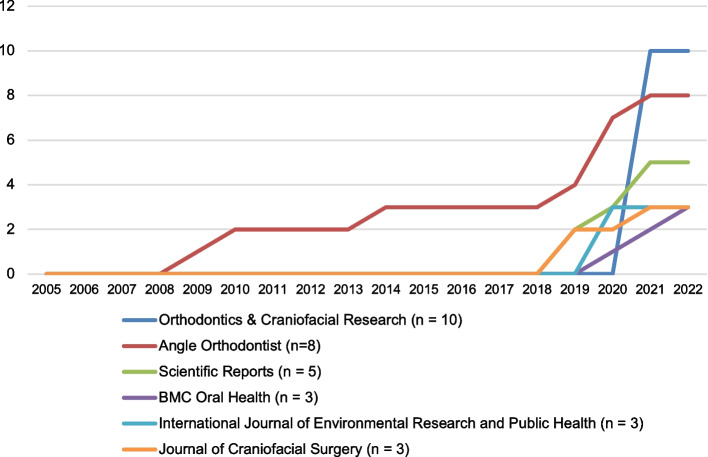
Publication trend in the top six journals with the highest number of publications

Figure 2 presents the top six influential journals with the highest number of cited articles in the field of AI in orthodontics and orthognathic surgery, each having published three or more articles. The journal with the highest number of articles is *Orthodontics & Craniofacial Research*, which has published a total of 10 articles, with a notable surge in publications in the year 2020. Following closely is *Angle Orthodontist* with eight articles.

### Author and institution profile

A total of 458 authors contributed to the 100 most-cited articles. Table 2 presents the top 10 authors who have made significant contributions, with each author having published 4 articles. Remarkably, among the top 10 authors, six are affiliated with South Korean institutions. A total of 180 universities have contributed to the top 100 articles. Among them, 17 institutions have made notable contributions with five or more articles each, as summarized in Table 3. Seoul National University stands out as the most productive institution, with 25 publications, closely followed by Korea University and Yonsei University, each with 13 publications. It is noteworthy that 14 out of the top 17 institutions are located in Asian countries, specifically South Korea, China, and Japan.

**Table 2:** Top 10 authors with the highest number of publications

Authors	Publications	Affiliations	Country
Seung-Hak BAEK	4	Seoul National University	South Korea
Richard E DONATELLI	4	University of Florida College of Dentistry	USA
Hye-Won HWANG	4	Seoul National University	South Korea
Reinhilde JACOBS	4	University Hospitals Leuven	Sweden
Min-ji KIM	4	Ewha Womans University	South Korea
Sang-Hwy LEE	4	Yonsei University	South Korea
Shin-Jae LEE	4	Seoul National University	South Korea
Jun-Ho MOON	4	Seoul National University	South Korea
Adriaan VAN GERVEN	4	Relu BV	Belgium
Holger WILLEMS	4	Relu BV	Belgium

**Table 3:** Top 17 institutions with the highest number of publications

Institutions	Country	Publications
Seoul National University	South Korea	25
Korea University	South Korea	13
Yonsei University	South Korea	13
Sichuan University	China	12
The University of North Carolina	United States	11
Jeonbuk National University	South Korea	10
Peking University	China	10
Osaka University	Japan	9
Wonkwang University	South Korea	6
Chang Gung University	Taiwan	5
Ewha Womans University	South Korea	5
Katholieke Universiteit Leuven	Belgium	5
Kyung Hee University	South Korea	5
Shahid Beheshti University of Medical Sciences	Iran	5
University of Hong Kong	Hong Kong SAR	5
University Hospital Leuven	Belgium	5
Zhejiang University	China	5

### Country profile and collaborative relationship

**Fig. 3:**
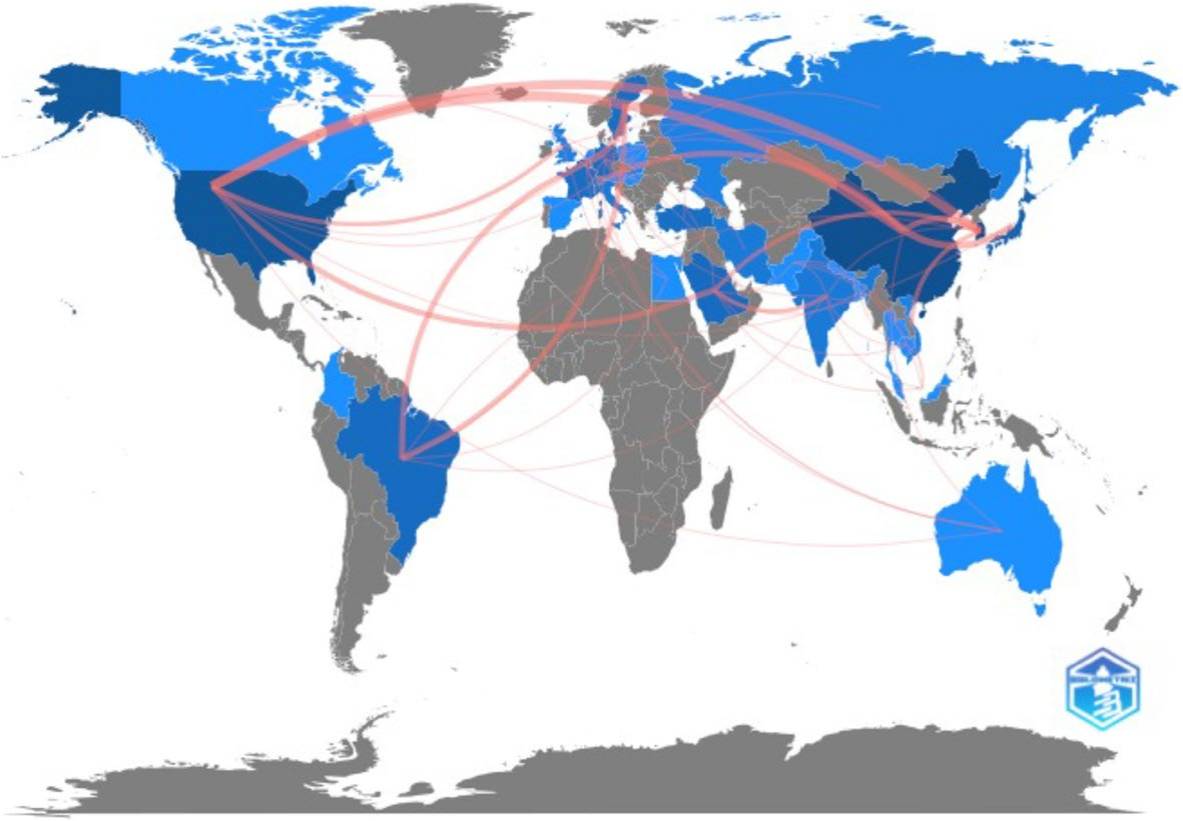
Collaboration network between countries. The size of the line is proportional to the number of articles collaborated between each country

The top 100 AI articles had corresponding authors from 24 different countries. Among these, South Korea had the highest number of publications (28) and citations (595), followed by China with 16 publications and 373 citations (Table 4). Notably, Germany exhibited the highest citation/publication ratio of 49. Furthermore, Fig. 3 presents the collaboration map, which illustrates the collaborative relationships between countries, emphasizing notable collaborations between the United States, South Korea, and China. Specifically, the United States has the highest number of collaborations with China (8) and South Korea (7), followed by Belgium and Sweden (4).

**Table 4:** Countries with more than one publication (only corresponding authors were considered)

Country	Citations	Publications	Citation/ publication ratio
South Korea	595	28	21.2
China	373	16	23.3
USA	248	7	35.4
Japan	127	7	18.1
Turkey	106	5	21.2
France	98	5	19.6
Germany	98	2	49
Belgium	94	5	18.8
Saudi Arabia	68	3	22.7
Switzerland	68	2	34
Iran	41	3	13.7
Italy	40	3	13.3
Brazil	37	2	18.5

### Keyword and classification of study domains

**Fig. 4:**
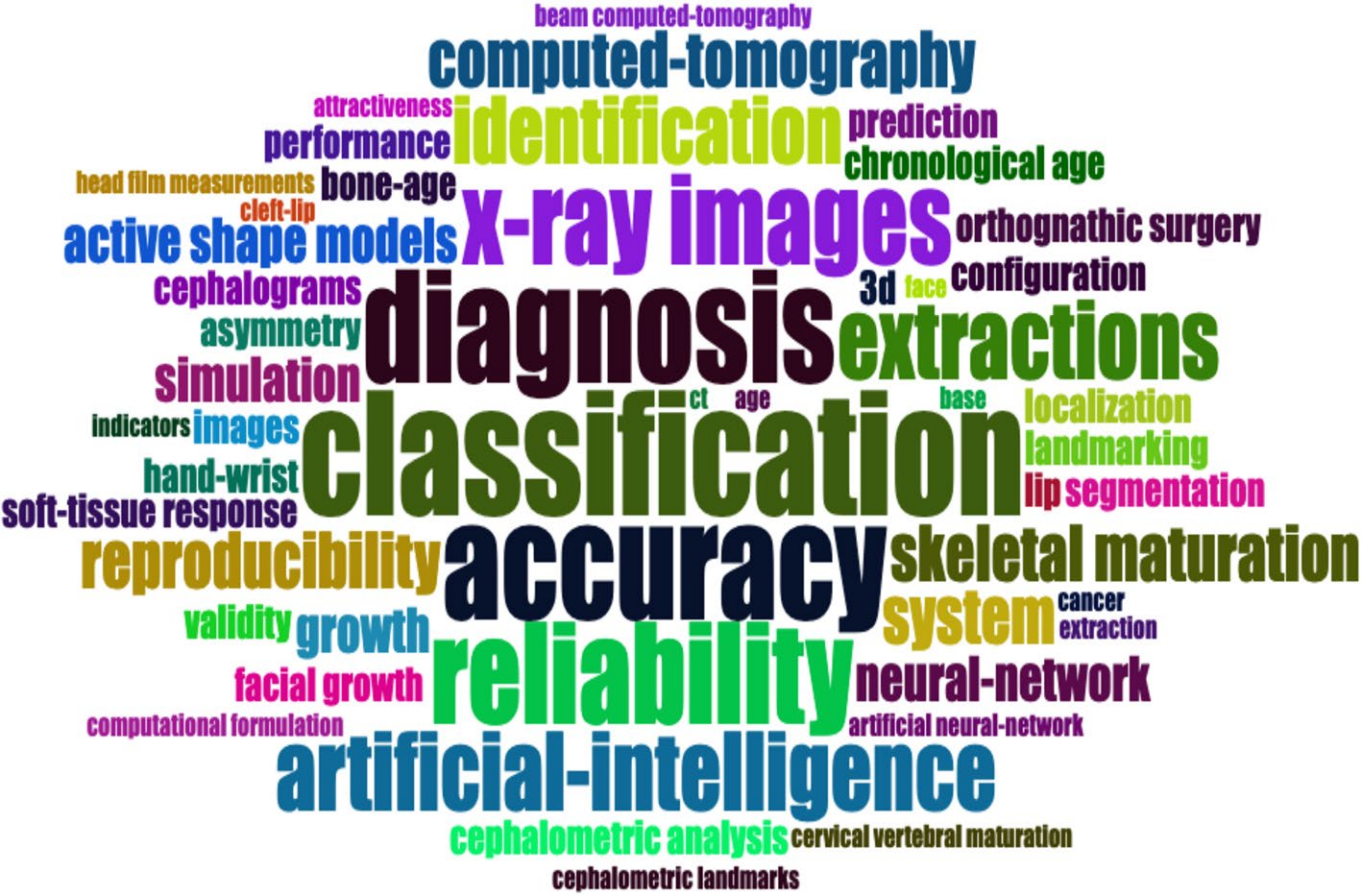
Keyword cloud of the top 100 most-cited articles

The keyword cloud of the articles in the collection is presented in Fig. 4. The most frequently referenced keyword is "classification." Furthermore, keywords such as "accuracy," "diagnosis," "reliability," and "x-ray images" also frequently referenced. The classification of study domains revealed that nearly half (*n* = 42) of the studies in the top 100 most-cited articles focused on automated cephalometric landmarking and/or analysis in two-dimensional (2D) cephalograms and three-dimensional (3D) CBCT images (Table 5). The application of automated landmark identification and analysis was more prevalent in 2D cephalograms compared to 3D imaging. Approximately one-third of the articles (*n* = 34) focused on diagnosis and treatment planning. Ten studies were dedicated to the assessment of growth and development. However, only two papers focused on AI in treatment process monitoring.

**Table 5:** Classification of top 100 articles by study domains

**Domains**	Number of studies
**1. Diagnosis and treatment planning**	**34**
a. Assessment of orthodontic extraction	2
b. Evaluation of facial attractiveness	2
c. Classification of skeletal patterns	5
d. Diagnosis and planning of orthognathic surgery	6
e. Assessment of orthodontic/orthognathic surgery need	6
f. Prediction of size of unerupted canine and premolar	1
g. Prediction of facial morphology after orthognathic surgery and orthodontic treatment	5
h. Evaluation of facial symmetry	2
i. Prognosis prediction for Class III malocclusion treatment	1
j. Detection of early signs of gingivitis	1
k. Palatal shape analysis	1
l. Detection of supernumerary teeth	1
m. Prediction of the eruption of the third molar	1
**2. Automated cephalometric landmarking and/or analysis**	**42**
a. 2D cephalograms (lateral and posteroanterior)	26
b. 3D imaging	16
**3. Assessment of growth and development**	**10**
a. Cervical vertebra maturation	9
b. Hand-wrist maturation	1
**4. Miscellaneous**	**14**
a. Review	7
b. Systematic review	2
c. Scoping review	3
d. Treatment process monitoring	2

## Discussion

The current study identifies and analyzes the top 100 most-cited articles pertaining to AI in orthodontics and orthognathic surgery, with the purpose of assisting future researchers in identifying emerging trends and areas that require further impactful research. Furthermore, this study acknowledged the authors, institutions, and sources that have made significant contributions to the advancement of AI in orthodontics and orthognathic surgery. The rationale behind combining orthodontic and orthognathic surgery in this study is their close association, as both fields can benefit from the application of AI as a valuable diagnostic tool.

As illustrated in Fig. 1, there is a remarkable exponential increase in AI publications from 2019 onwards. This growth aligns with McKinsey's Global Survey results, which revealed a significant rise in AI adoption from 20% in 2017 to 50% in 2022 [19]. While it is challenging to pinpoint the exact reason for the sudden surge of interest in AI research in orthodontics and orthognathic surgery, one possible explanation is the increasing adoption of AI technology in clinical practices. A similar trend was observed in the field of medical imaging, where the use of deep learning networks rapidly gained traction since its publication in 2016, peaking in 2020 [20].

Among the 100 most-cited articles, *Orthodontics & Craniofacial Research* emerged as the leading publication source, with the highest number of articles (*n* = 10). This achievement can be attributed to the journal's publication of a special issue on "Artificial intelligence and machine learning in orthodontics" in 2021, which had a significant impact on the orthodontics and orthognathic surgery communities. As a result, *Orthodontics & Craniofacial Research* surpassed *Angle Orthodontist* as the primary publication source for the top 100 AI articles. This observation underscores the potential influence of publishing special issue journals on research trends and demonstrates how such initiatives can help focus on emerging interests in the field and highlight new research applications.

According to our findings, six South Korean authors were among the top 10 contributors, and five South Korean institutions contributed significantly to the 100 most-cited AI articles. Notably, two of the top 10 authors, coming from non-orthodontic or orthognathic surgery backgrounds and affiliated with Relu BV, a dental software company specializing in AI algorithms for automating digital dental treatment planning, bring valuable perspectives from technological and engineering fields to advance our understanding of AI in this domain. Furthermore, South Korea leads with the highest number (*n* = 28) of publications among the 100 most-cited articles, closely followed by China (*n* = 16), Japan (*n* = 7), and United States (*n* = 7). These findings differ slightly from a previous study, which reported China as having the highest number of published studies on AI in dentistry, followed by the USA, South Korea, and India [21]. The differences in findings could be attributed to our analysis considering the country of origin of the corresponding author and focusing specifically on the field of orthodontics and orthognathic surgery, rather than encompassing the entire dental field. Moreover, Germany exhibited the highest citation-to-publication ratio among the top 100 cited AI articles, highlighting the impact and influence of their two publications. However, it is important to note that citation numbers solely reflect popularity and influence, and may not necessarily indicate the quality of the research [22]. Therefore, it is crucial to recognize as a limitation of the bibliometric study that the assessment of article quality was not conducted.

Unlike the country profile, which solely focuses on the nationality of the last corresponding author, the analysis of collaboration in our study encompasses the profiles of all co-authors. Our findings highlight that the most active international collaborations are observed between the USA, China, and South Korea. Furthermore, active collaborations are also observed between Brazil, Sweden, and Belgium. These variations in collaboration patterns between countries may be attributed to differences in research interests, funding resources, and languages, as suggested by previous studies [12].

AI can be categorized into two main types: narrow AI and strong AI. Narrow AI utilizes learning algorithms to solve specific tasks, and the knowledge acquired is not transferable to other tasks. On the other hand, strong AI refers to AI systems with human-level intelligence, possessing awareness and behavior similar to humans [23]. Strong AI aims to create a multi-task algorithm to make decisions in multiple fields. However, the development of strong AI raises ethical considerations and potential risks [24]. Currently, there are no strong AI applications in dentistry [25]. Table 5 displays the domains of the top 100 most-cited studies related to AI in the fields of orthodontics and orthognathic surgery. It is revealed that these studies primarily fall into categories such as automated imaging assessment (42%) and the application of AI in aiding diagnosis and treatment planning (33%). Cephalometric analysis, although an essential process in orthodontics, is prone to human error when performed manually [26, 27]. To address this, machine learning AI technologies such as convolutional neural networks have been developed for graphic image analysis. It utilizes multiple-layered connections to pass distinctive features to subsequent layers [28]. These advancements have facilitated the automation of cephalometric tracing and analysis, offering several benefits, including reduced human labor and decreased errors [29]. Popular keywords found in the studies included "deep learning," "machine learning," "convolutional neural network," and "automated identification,", highlighting the significant interest in these AI technologies. Notable examples of automated tracing and landmark identification systems, such as CephX (ORCA Dental AI, Israel) and WebCeph (AssembleCircle, South Korea) [30, 31]. Furthermore, AI algorithms can remove noise, enhance contrast and fine-tune images to provide dentists with clearer radiographs [32]. Regarding the accuracy of AI-facilitated cephalometric landmark detection, a systematic review conducted by Schwendicke et al. revealed high accuracy in detecting cephalometric landmarks in both 3D and 2D imaging [1]. However, there was notable heterogeneity in detection accuracy between individual landmarks. In 3D imaging, the proportion of landmarks detected within a 2 mm threshold was higher (0.870) compared to 2D imaging (0.792). Furthermore, a more recent study demonstrated significant accuracy in AI-facilitated 3D cephalometric landmarking, with a mean difference of 2.44 mm (95% CI 1.83–3.05) between automated and manual landmarking [33]. Interestingly, such discrepancy showed a decreasing trend over the years, suggesting advancements and improvements in AI technology.

Furthermore, it is noteworthy that the training and testing sample sizes varied significantly among the different studies, ranging from 18 to 20480 for the training sample size and 6 to 5120 for the testing sample size. Interestingly, our analysis identified a significant positive correlation between the testing sample sizes and the citation counts indicating that papers with larger testing samples tend to receive more citations. Furthermore, earlier publication dates were associated with higher citation counts. However, it is essential to consider that other factors, such as the research topic, journal, authors, and institutions, may also influence the citation counts.

One limitation of this study is the relatively recent emergence of research on the application of AI in orthodontics and orthognathic surgery, which may contribute to a lower number of citations compared to more established dental topics with a longer research history. Additionally, while the authors' profiles were analyzed to explore collaboration between countries, the specific level of contribution from each author could not be determined, potentially leading to an overestimation of collaboration. Furthermore, the quality of the included studies was not sufficiently assessed, and the level of evidence may be varied. Lastly, the use of the WoS Core Collection database, which primarily includes English-language articles, may have resulted in the exclusion of impactful studies published in other languages.

## Conclusion

- 1. The top 100 most-cited articles in AI in orthodontics and orthognathic surgery were authored by 458 researchers from 180 institutions across 24 countries, with a significant surge in publications starting from 2019.
- 2. South Korea has the highest number of publications and citations, followed by China and the United States. Seoul National University had the highest number of publications among the top 100 most-cited articles. Furthermore, the United States, China, and South Korea have been actively engaged in international collaborations.
- 3. The majority of the articles were focused on automated imaging assessment and the application of AI in aiding diagnosis and treatment planning.

### Supplementary Information

**Additional file 1. **The full list of the top 100 most-cited articles in orthodontics and orthognathic surgery.

## Data Availability

The data used and analyzed during the current study are available from the corresponding author on reasonable request.

## Abbreviations

AI: Artificial intelligence
WOS: Web of Science
2D: Two-dimensional
3D: Three-dimensional
